# Tumeur de vessie chez le sujet jeune: à propos de 36 cas

**DOI:** 10.11604/pamj.2014.18.52.4553

**Published:** 2014-05-15

**Authors:** Mouad Statoua, Jihad El Ghanmi, Tarik Karmouni, Khalid El Khader, Abdellatif Koutani, Ahmed Iben Attya

**Affiliations:** 1 Service d'Urologie B, CHU IBN Sina, Rabat, Maroc

**Keywords:** Tumeur de vessie, sujet jeune, épidémiologie, histologie, traitement, évolution, pronostic, ballder tumor, young patients, epidemiology, histology, treatment, evolution, prognosis

## Abstract

Les tumeurs de vessie sont rares avant 40 ans et ne représentent que 1 à 4% de l'ensemble de ces tumeurs. Notre étude s'est intéressée au profil épidémiologique, caractère histologique ainsi que le profil évolutif. Nous avons réalisé une étude retrospective sur une durée de 10 ans entre mars 2004 et mars 2014, nous avons traité 850 patients pour tumeur de vessie dont 36 était âgé de moins de 40 ans. Le diagnostic était posé devant des arguments cliniques, radiologiques et endoscopiques, la cystoscopie avec résection tumorale a précisé la nature histologique et son grading ainsi que le degré d'infiltration. 23 patients avaient une tumeur de vessie non infiltrant le muscle et 13 avaient une tumeur infiltrant le muscle. Le taux de récidive global est de l'ordre de 36,4%, 22% pour la tranche d’âge 20-30 ans et 46% pour la tranche d’âge 31-40 ans. Le risque de progression et de décès est respectivement de 8% et de 6,1%. Il ressort que les tumeurs superficielles ont une meilleure évolution chez les sujets de moins de 30 ans. Les tumeurs infiltrantes sont plus fréquentes et souvent évoluées suggérant un potentiel évolutif particulier. Leur pronostic est fonction du stade tumoral, sans corrélation avec l’âge.

## Introduction

Les tumeurs de vessie sont fréquentes. Elles représentent le deuxième cancer urologique. Elles sont classiquement considérées comme une maladie du sujet âgé. Nous assistons cependant à une atteinte de plus en plus croissante des sujets jeunes, du fait certainement d'une influence de l'environnement et d'une modification des habitudes de vie. Le tabac est un facteur important dans la genèse de ces tumeurs, mais d'autres facteurs peuvent intervenir notamment d'ordre héréditaire. Leur profil évolutif est encore mal connu. Nous avons mené au sein de notre formation une étude sur la prévalence de la tumeur de vessie chez les sujets de moins de 40 ans en essayant de préciser les facteurs de risque dans ce groupe de population, les caractéristiques de cette tumeur ainsi que de tenter de déterminer le profil évolutif après traitement.

## Méthodes

Sur une période de dix ans s’étalant entre mars 2004 et mars 2014, nous avons pris en charge 36 patients qui présentaient une tumeur de vessie avec un âge moyen de 33 ans (20-40 ans, notre étude s'intéressant a cette tranche d’âge), on a noté une prédisposition masculine (29 hommes pour 7 femmes), le tabac était le facteur de risque majeur avec une moyenne de consommation de l'ordre de 10 paquets/année, ainsi que l'exposition professionnelle rapporté chez 2 cas.

Le signe clinique révélateur était l'hématurie observée chez 90% des patients souvent macroscopique totale ou terminale, les examens biologiques réalisés appréciaient le retentissement de la pathologie et portaient sur le dosage de l'hémoglobine qui a objectivé une anémie chez 14 patients nécessitant des transfusions et l’évaluation de la fonction rénale qui a mis en évidence trois cas d'insuffisance rénale.

**Figure 1:**
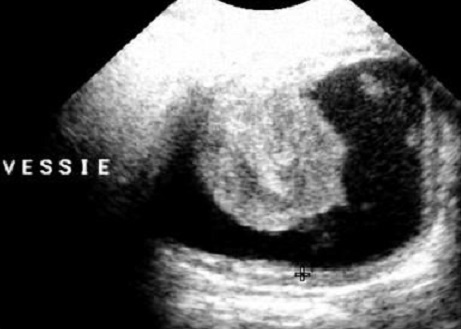
Echographie vésicale montrant un processus intravésical

L’échographie réno-vésicale était l'examen radiologique utilisé pour objectivé le processus intravésicale ainsi que l’évaluation du retentissement du processus sur le haut appareil urinaire (Figure 1).

**Figure 2:**
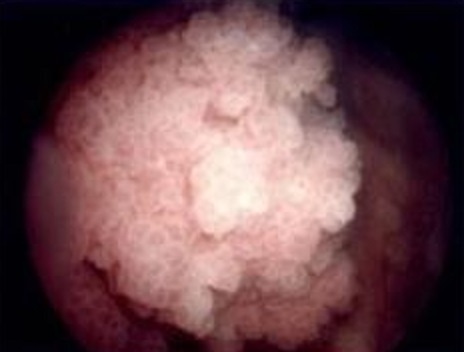
Aspect macroscopique d'un polype vésical lors d'une cystoscopie

Une cystoscopie a été réalisée chez tous nos patients avec une résection profonde emportant la base tumorale pour objectiver le degré d'infiltration tumorale, après analyse histologique tous nos patients avaient un carcinome urothélial qui était superficiel chez 23 patients et profond chez 13 (Figure 2).

## Résultats

**Figure 3:**
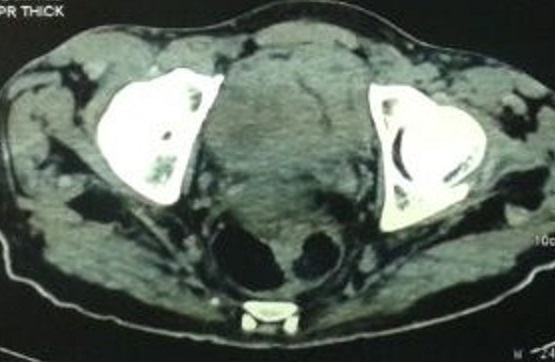
Image scannographique montrant un processus intravésical

**Figure 4:**
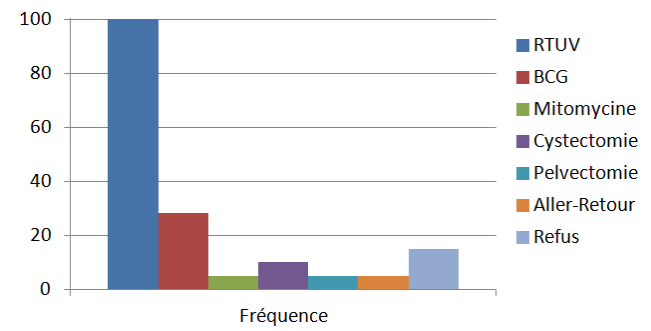
Diagramme résumant les modalités de prise en charge

Les patients qui présentaient une tumeur superficielle ont été classé en groupe pronostic ainsi 11 patients été classé à faible risque vu qu'ils avaient une localisation unique, inférieure à 3 cm et dont le résultat anatomopathologique était Ta bas grade, les patients classés à haut risque de progression qui était au nombre de dix et qui avaient des tumeurs multifocale et/ou un haut grade sur le staging cytonucléaire ont bénéficié d'un traitement adjuvant à base de BCG thérapie et enfin deux patients qui avait des localisations supérieures à 3 cm avec un résultat anapathologique en faveur d'un carcinome urothéliale Ta bas grade ont bénéficié d'instillations endovésicale de la mitomycine C, on avait proposé au patients classé bas grade un complément thérapeutique à base de mitomycine C ou BCG thérapie mais par défaut de moyen ont préféré une surveillance qui est le standard dans ce cadre, pour les patients présentant une tumeur de vessie infiltrante après un bilan d'extension consistant en la réalisation d'un scanner thoraco-abdomino-pelvien (Figure 3), une cystectomie était réalisée chez 4 patients, 2 pelvectomie antérieure, 2 aller retour du fait du caractère fixe et inextirpable des masses et cinq autres patients ont refusé le geste radicale (Figure 4).

La surveillance consistait en la réalisation de cystoscopie de contrôle tous les 6 mois chez les patients présentant des tumeurs superficielles, on a noté la récidive chez 4 patients qui n'ont pas bénéficié de traitement adjuvant mais sans progression tumoral vers le caractère infiltrant et sur le même caractère histologique après la nouvelle résection, pour les patients bénéficiant de gestes radicaux un contrôle trimestrielle de la fonction rénale et un scanner de contrôle fut réalisé à 6 mois de l'intervention ainsi que l'appréciation de la tolérance du type de dérivation qui était des enterocystoplasties de remplacement chez les 4 hommes et des dérivations externe type bricker chez les 2 femmes.

## Discussion

Les tumeurs de vessie sont très rares chez le sujet de moins de 40 ans. Le premier cas fut rapporté en 1872 par Smythe [1]. Plusieurs études montrent que les tumeurs vésicales chez le sujet jeune ne représentent que 0,4 à 1% de l′ensemble des tumeurs de vessie diagnostiquées [1–5] (4,2% dans notre série). On retrouve dans la littérature, comme chez l′adulte de plus de 40 ans, une nette prédominance masculine diversement appréciée selon les auteurs, le sexe ratio variant de 3,6 à 9 hommes pour une femme [2–6]. Si l′hématurie macroscopique est le symptôme révélateur le plus souvent constaté (91% dans notre série), le diagnostic doit être également évoqué en cas de signes irritatifs vésicaux et devant des lombalgies. Parmi les facteurs de risque reconnus de tumeur urothéliale, le tabagisme reste prépondérant, comme chez le sujet plus âgé. Ce risque est classiquement lié au nombre de paquet/année et au degré d′inhalation de la fumée. Il est de l'ordre de 32% dans notre série, cependant c'est difficile chez le sujet jeune, notamment avant 30 ans d′affirmer le rôle nocif du tabac compte tenu de la faible durée d′exposition dans notre série. Quoique le rapport de l'OMS 2007 montre que 15,5% des fumeurs sont âgés de 13 à 15 ans et 24% des jeunes fumeurs ont commencé avant l’âge de 10 ans. Dans notre étude, deux patients présentaient un facteur de risque professionnel (peintres). Benton et Henderson [1, 7], sur une étude de 9 jeunes gens présentant un carcinome urothélial, constatent à six reprises une exposition aux peintures, solvants ou produits chimiques. La durée de cette exposition varie entre 3 et 11 ans. La prédisposition héréditaire aux néoplasies vésicaux fut évoquée pour la première fois par Fraumeni [1, 8] en 1967. Plusieurs études rétrospectives ont constaté un taux plus élevé d′anomalies sur les chromosomes 7 et 17 dans les tumeurs de vessie évolutives du sujet âgé [9]. Iori [10] et Linn [11] retrouvent un taux élevé de mutation sur ces 2 chromosomes dans leur population de sujets jeunes. Ainsi la découverte d'une tumeur de vessie chez un sujet jeune doit conduire à une enquête familiale et génétique afin de dépister les familles à risque et assurer une surveillance urologique rigoureuse.

Une analyse des différentes publications objective la fréquence des tumeurs superficielles ainsi Kutarski [12], dans une revue de littérature, s′est intéressé à la variation du stade histologique en fonction de l′âge et a constaté que le taux de tumeur Ta est de 77% au cours de la 4ème décade, avant 20 ans, ce taux atteint 95%. Concernant les tumeurs infiltrantes, les résultats sont là encore très variables en fonction des séries. Ainsi Javadpour [3] et Kutarski [12] ne retrouvent aucune tumeur infiltrante dans leur étude. A l′inverse, Aboutaieb [1, 13] isole 14 cas de tumeurs infiltrantes sur 25 tumeurs urothéliales.

En ce qui concerne le risque de récidive et de progression les différentes publications divergent sur ce profil évolutif néanmoins on a constaté que le taux de récidive des tumeurs superficielles de stade Ta bien et moyennement différenciées (G1 et G2) chez le sujet âgé varie entre 50 et 55% [12]. Par contre au cours de la 3ème décade, le taux de récidive n′est plus que de 17% et avant 20 ans, ce taux est à 5%. En ce qui concerne le taux de progression, il est de 17% au cours de la quatrième décade. Ce chiffre n′est plus que de 4% au cours de la 3ème décade. Aucun cas de progression n′a été rapporté avant 20 ans. Dans notre série le taux de récidive global est de l'ordre de 36,4%, 22% pour la tranche d’âge 20-3

## Conclusion

Les tumeurs de vessie restent rares chez le jeune. Le tabac est un facteur de risque important. Le profil évolutif des tumeurs superficielles nous a semblé différent avant et après 30 ans. Avant 30 ans, l’évolution est favorable avec peu de récidives. Après 30 ans, le risque de récidive et de progression tumorale semble comparable à celui du sujet âgé. Le pronostic des tumeurs infiltrantes est par contre habituellement très sombre révélant un potentiel agressif particulier.

## References

[1] Blanchard JM, Graziana JP, Bonnal JL, Biserte J, Mauroy B (2003). Tumeurs de vessie du sujet jeune: à propos de 26 cas, comparaison aux données de la littérature. Prog Urol..

[2] Cherrie RJ, Lindner A, Dekernion JB (1982). Transitional cell carcinoma of bladder in first four decades of life. Urology..

[3] Javadpour N, Mostofi FK (1969). Primary epithelial tumors of the bladder in the first two decades of life. J Urol..

[4] Johnson DE, Hillis S (1978). Carcinoma of the bladder in patients less than 40 years old. J Urol..

[5] Mc Carthy JP, Gavrell GJ, Leblanc GA (1979). Transitional cell carcinoma of bladder patients under thirty years of age. Urology.

[6] Alcaraz A, Talbot-Wright R, Samson R, Mestres CA (1991). Vesical tumors in patients under 25 years of age. Eur Urol..

[7] Benton B, Henderson E (1973). Environmental exposure and bladder cancer in young males. J Nat Cancer Institute..

[8] Fraumeni JF, Thomas LB (1967). Malignant bladder tumors in a man and his three sons. JAMA.

[9] Benton B, Henderson E (1973). Environmental exposure and bladder cancer in young males. J Nat Cancer Institute.

[10] Sidransky D, Von Eschenbach A, Tsai YC, Jones P, Summerhayes I (1991). Identification of p53 gene mutations in bladder cancers and urine samples. Science..

[11] Iori F, De Dominicis C, Liberti M, Frioni D, Vahedi M (2001). Superficial bladder tumors in patients under 40 years of age: clinical, prognostic and cytogenetic aspects. Urol Int..

[12] Linn JF, Sesterhenn I, Mostofi FK, Schoenberg M (1998). The molecular characteristics of bladder cancer in young patients. J Urol..

[13] Kutarski PW, Padwell A (1993). Transitional cell carcinoma of the bladder in young adults. Br J Urol..

[14] Aboutaieb R, Dakir M, Sarrf I (1998). Les Tumeurs de vessie chez le sujet jeune. Prog Urol..

